# Simulation as tool for evaluating and improving technical skills in laparoscopic gynecological surgery

**DOI:** 10.1186/s12893-019-0610-9

**Published:** 2019-10-16

**Authors:** Paolo Mannella, Elisa Malacarne, Andrea Giannini, Eleonora Russo, Marta Caretto, Francesca Papini, Maria Magdalena Montt Guevara, Federica Pancetti, Tommaso Simoncini

**Affiliations:** Department of Clinical and Experimental Medicine, University of Pisa, Via Roma 67, 56126 Pisa, Italy

**Keywords:** Simulation, Laparoscopy, OSATS, Training

## Abstract

**Background:**

Simulation in laparoscopic surgery is nowadays recognized as a valid instrument for learning and training surgeons in different procedures. However, its role as evaluation test and self-assessment tool to verify basic surgical skills is still under discussion.

**Methods:**

Thirty-three residents in obstetrics and gynecology at University of Pisa, Italy were recruited, and they received a simulation program consisting of 5 tasks. They had to perform basic laparoscopic surgery maneuvers as creating pneumoperitoneum, positioning trocars under vision, demonstrating the appropriate use of dominant and non-dominant hand and making single stitch and knot. They were evaluated with a modified OSATs scale.

**Results:**

Senior trainees had better score than junior trainees (*p* value< 0,005) and after different sessions of simulation scores of both groups significantly improved (*p* < 0,001), especially for the junior group. All the trainees reported self-assessments that matched with the evaluation of external observers demonstrating the importance of simulation also as auto-evaluation test.

**Conclusions:**

In this study, we demonstrated the role of simulation as powerful tool to evaluate and to self-assess surgical technical skills and to improve own capacities, with the use of a modified OSATs scale adapted to specific exercises.

## Background

Surgical training is one of the most important aspects in different medical specialties. Trainees can obtain competences in traditional and minimally invasive surgery only after years of practice and sacrifices. Traditionally, one of the main methods of surgical residency training remains an intensive internship in the operating room, although it presents several negative aspects including potential risks to the patient safety [1] and the need for trainees to spend many hours in the operating theatre before achieving good results, with limited training opportunities due to the lack of time and to the many professional activities that must be performed [2]. To overcome difficulties about this “learning-by-doing” approach, a lot of simulators and box trainers have been tested in the last decades to evaluate their effectiveness for surgery training, demonstrating their ability to improve technical skills, operative performance and coordination [3–5] and therefore simulation programs are now considered a key role in the surgical learning process. The American Board of Surgery in 2008 announced that, among the necessary requisites to complete general surgery residency in the United States, the Fundamentals of Laparoscopic Surgery (FSL) were mandatory; the goal of this program is to allow residents to learn and practice technical skills and then test them to ensure an appropriate required skills level [6]. Also, the program of the OBGYN residency could benefit from training models. Several studies demonstrated the role of simulation to improve obstetric skills in specific clinical situation such as shoulder dystocia, vaginal delivery for breech presentation or vacuum extraction [7–10]. The aim of this study is to demonstrate the role of simulation in laparoscopy on improving technical skills in trainees in obstetrics and gynecology and as evaluation tool to self-assess own capacities with the use of a modified OSATs scale.

## Methods

Thirty-three residents (post-graduate training year, PGY, 1–5) from the Division of Obstetrics and Gynecology of University of Pisa, Italy, performed five simulated surgical procedures and they were evaluated by an external observer who is an expert surgeon with a high experience in minimally invasive surgery. All the procedures were selected to assess laparoscopic skills and they showed a level of increasing difficulty using a high-fidelity simulation platform, the Simsei training system (© 2018 Applied Medical). The five-tasks for each station were:

1. creating pneumoperitoneum and positioning trocars under vision, using a first entry kit that reproduced the characteristics of the abdominal wall
2. moving six pegs on a platform, from right to left and from left to right using dominant and non-dominant hand
3. changing the shape of a rubber band on a platform with spikes using dominant and non-dominant hand
4. cutting precisely a circle printed on a dual layer gauze which was laterally fixed with supports
5. making single stitch/knot on a silicon suture-pad that simulated tissues.

Trainees performed all the procedures several times at different moment of the day, by their own.

In the first phase, procedures were executed without any information about the “correct” type of execution. This phase was permitted to make trainees confident with simulation. At the beginning, trainees received only indications concerning the exercises and then they started doing procedures.

Each procedure was evaluated by four expert surgeons of our department, that were randomly assigned and each trainee filled a self-evaluation test. After that, the correct execution of each task was shown by expert surgeons to the trainees and, after 2 h of training, they repeated all the five procedures again and a new evaluation was performed.

Procedures were executed in the same order, from exercise number one to exercise number five. Time used to perform the procedure was evaluated only in the fourth station.

Faculty members, during the second examination, could not see previous results to avoid any influence in the judgment.

Assessments were given using OSATS (Objective Structured Assessment of Technical Skills) (Table 1) that were specifically adapted for each station to evaluate surgical abilities for each procedure. OSATS presented scores ranging from 1 to 5, with minimum and maximum score of 3 and 15 for the first station, 4 and 20 for the second, third, and fourth stations, 5 and 25 for the fifth station.

**Table 1:** Objective Structured Assessment of Technical Skills assessment test: each trainee was evaluated in five different task with a score from 1 (low) to 5 (high)

TASK 1: creating pneumoperitoneum and positioning trocars under vision					
Time and motion	1	2	3	4	5
	Many unnecessary moves		Efficient time/motion but some unnecessary moves		Economy of movement and maximum efficiency
Knowledge of instruments	1	2	3	4	5
	Frequently asked for the wrong instrument or used an inappropriate instrument		Knew the names of most instruments and used appropriate instrument for the task		Obviously familiar with the instruments required and their names
Instruments handling	1	2	3	4	5
	Repeatedly makes tentative or awkward moves with instruments		Competent use of instruments although occasionally appeared stiff or awkward		Fluid moves with instruments and no awkwardness
TASK 2: moving six pegs on a platform, from right to left and from left to right					
Time and motion	1	2	3	4	5
	Many unnecessary moves		Efficient time/motion but some unnecessary moves		Economy of movement and maximum efficiency
Instruments handling (dominant hand)	1	2	3	4	5
	Repeatedly makes tentative or awkward moves with instruments		Competent use of instruments although occasionally appeared stiff or awkward		Fluid moves with instruments and no awkwardness
Instruments handling (non-dominant hand)	1	2	3	4	5
	Repeatedly makes tentative or awkward moves with instruments		Competent use of instruments although occasionally appeared stiff or awkward		Fluid moves with instruments and no awkwardness
Synchronization between the hands	1	2	3	4	5
	No coordination between the movements of the two hands		At times there is coordination between the movements of the two hands		Maximum synchronization between the hands
TASK 3: changing the shape of a rubber band on a platform with spikes					
Time and motion	1	2	3	4	5
	Many unnecessary moves		Efficient time/motion but some unnecessary moves		Economy of movement and maximum efficiency
Instruments handling (dominant hand)	1	2	3	4	5
	Repeatedly makes tentative or awkward moves with instruments		Competent use of instruments although occasionally appeared stiff or awkward		Fluid moves with instruments and no awkwardness
Instruments handling (non-dominant hand)	1	2	3	4	5
	Repeatedly makes tentative or awkward moves with instruments		Competent use of instruments although occasionally appeared stiff or awkward		Fluid moves with instruments and no awkwardness
Synchronization between the hands	1	2	3	4	5
	No coordination between the movements of the two hands		At times there is coordination between the movements of the two hands		Maximum synchronization between the hands
TASK 4: cutting precisely a circle printed on a dual layer gauze					
Time and motion	1	2	3	4	5
	Many unnecessary moves		Efficient time/motion but some unnecessary moves		Economy of movement and maximum efficiency
Instruments handling	1	2	3	4	5
	Repeatedly makes tentative or awkward moves with instruments		Competent use of instruments although occasionally appeared stiff or awkward		Fluid moves with instruments and no awkwardness
Respect for tissue	1	2	3	4	5
	Frequently used unnecessary force on tissue or caused damage by inappropriate use of instruments		Careful handling of tissue but occasionally caused inadvertent damage		Consistently handled tissues appropriately with minimal damage
Synchronization between the hands	1	2	3	4	5
	No coordination between the movements of the two hands		At times there is coordination between the movements of the two hands		Maximum synchronization between the hands
TASK 5: making single stitch/knot on a silicon suture-pad					
Time and motion	1	2	3	4	5
	Many unnecessary moves		Efficient time/motion but some unnecessary moves		Economy of movement and maximum efficiency
Instruments handling	1	2	3	4	5
	Repeatedly makes tentative or awkward moves with instruments		Competent use of instruments although occasionally appeared stiff or awkward		Fluid moves with instruments and no awkwardness
Respect for tissue	1	2	3	4	5
	Frequently used unnecessary force on tissue or caused damage by inappropriate use of instruments		Careful handling of tissue but occasionally caused inadvertent damage		Consistently handled tissues appropriately with minimal damage
Synchronization between the hands	1	2	3	4	5
	No coordination between the movements of the two hands		At times there is coordination between the movements of the two hands		Maximum synchronization between the hands
Flow of operation and forward planning	1	2	3	4	5
	Frequently stopped operating or needed to discuss next move		Demonstrated ability for forward planning with steady progression of operative procedure		Obviously planned course of operation with effortless flow from one move to the next

Tasks were the same for young and senior trainees and no differences on procedures were considered between trainees with higher experience in surgical room or different sub-specialties. This aspect is pivotal to consider. The training program of Department of Experimental and Clinical Medicine, Division of Obstetrics and Gynecology, University of Pisa, Italy, indeed provides a basic level of surgical competences for all the trainees during 5 years. Therefore, each resident, starting from the fourth year of residency, can decide which subspecialty focus on.

### Objective structured assessment of technical skills

Original OSATS model was developed in 1997 to evaluate general surgery residents performing operative tasks both on live anaesthetized animals and on bench models. The scoring system included three parts and it was used for each task. It consisted in: 1) a task specific check-list 2) a seven-item global rating scale 3) a pass/fail judgement [11]. In our study, we partially modified the original OSATS model (Table 1) to fit on our type of simulation model.

We organized 5 different tasks, which covered the basic skills to perform laparoscopic surgery. An external observer reported the competence of trainees with a score from 1 to 5, considering 5 as optimum.

In task 1, we evaluated the competence of trainees on creating pneumoperitoneum and positioning trocars correctly under vision. Then, in other tasks we evaluated other important aspects of mini-invasive surgery as instrument handling and synchronization between the hands (task 2 and 3), the careful handling of tissue and the cutting technique (task 4) and indeed, the making stitches and knots with laparoscopic instruments (task 5). For each task, we considered time and motion of trainees. Time was evaluated with a general consideration of the time of execution of each exercise. Only for task 4, which is considered the summary of different expertise and laparoscopic skills, we recorded the exact time (in seconds) occurred to complete the entire exercise.

### Statistical analysis

Statistical analysis was performed using GraphPad Prism 7 (GraphPad Software). Wilcoxon matched-pairs signed rank test was used to study time-operative outcomes in ex 4. In addition, two-way Anova was performed followed by Holm-Sidak’s multiple comparisons test. The values of *p* < 0.05 were considered significant. (*p < 0,05; **p < 0,01; ***p < 0,001).

## Results

### Simulation as tool to evaluate different competences of trainees

**Fig. 1:**
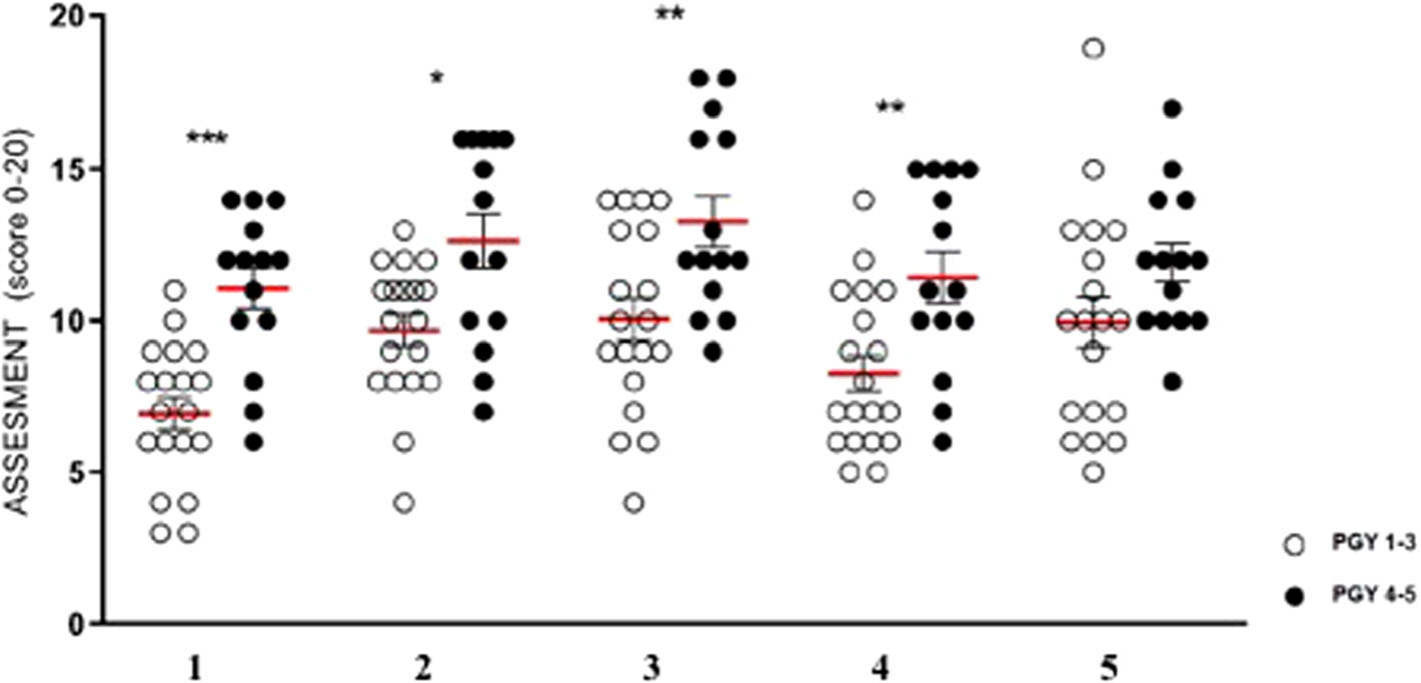
White spots: scores of junior trainees; Black spots: scores of senior trainees; *: *p* value < 0,05; **: p value < 0,01; ***: p value < 0,001)

Main scores, obtained before the simulation training, show similar results for all the stations (Fig. 1), with junior trainees (PGY 1–3) performing worse scores in comparison to senior trainees (PGY 4–5). However, this trend was statistically significant for the task 1–4 but not for the task 5 (Exercise 1: 6.947 vs. 11.07, *p* = 0.0004; Ex. 2: 9.684 vs. 12.64, *p* = 0.0196; Ex. 3: 10.05 vs. 13.29, *p* = 0.0084; Ex. 4: 8.263 vs. 11.43, *p* = 0.0104; Ex 5: 9.947 vs. 11.93, *p* = 0.2340).

### The use of simulator improves technical skills in each exercise

**Fig. 2:**
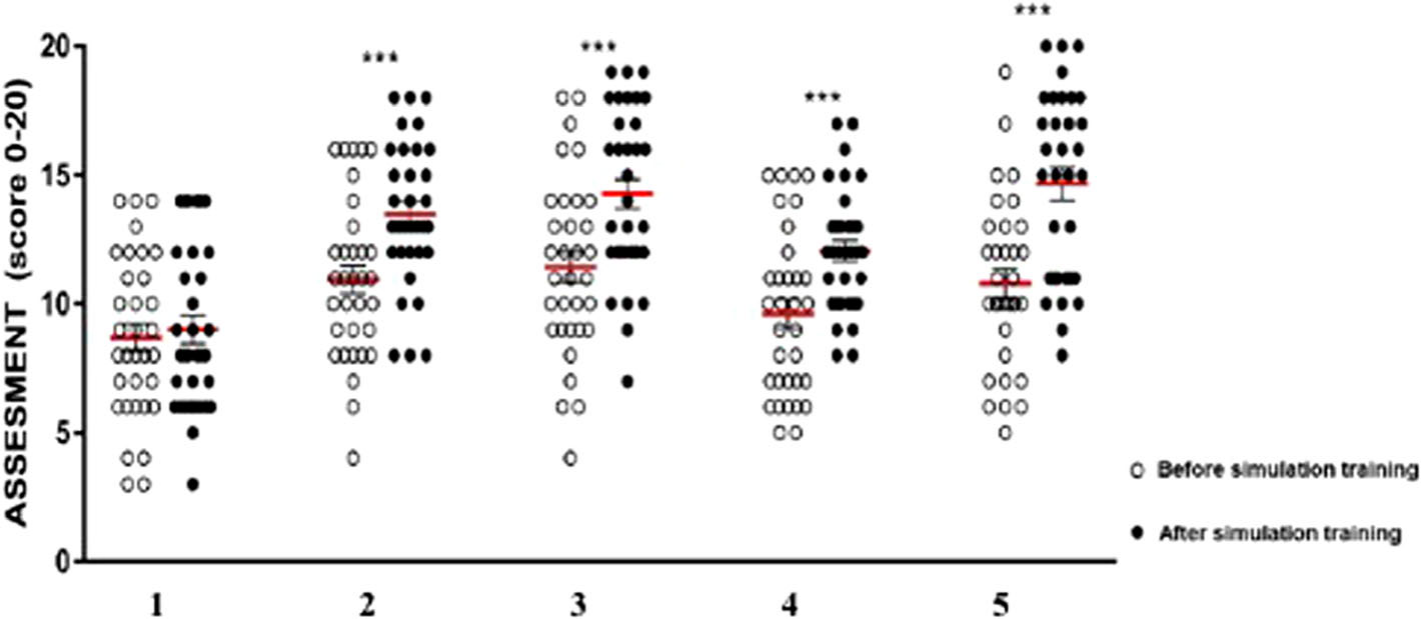
White spots: scores before simulation training; Black spots: scores after simulation training; ***: p value < 0.0001

Trainees improve their OSATS mean scores with the use of simulator. Except for exercise 1, the use of simulator improves the mean score of the other exercises (2–5) in a statistical significant way, as shown in Fig. 2 (Exercise 1: 8.697 vs. 9.03, *p* = 0.6686; Ex. 2: 10.94 vs. 13.48, *p* = 0.00119; Ex. 3: 11.42 vs. 14.27, *p* = 0.00029; Ex. 4: 9.606 vs. 12.06, *p* = 0.00176; Ex.5: 10.79 vs. 14.67, *p* < 0.00001).

### Simulation improves technical skill especially in “naïve” residents

**Fig. 3:**
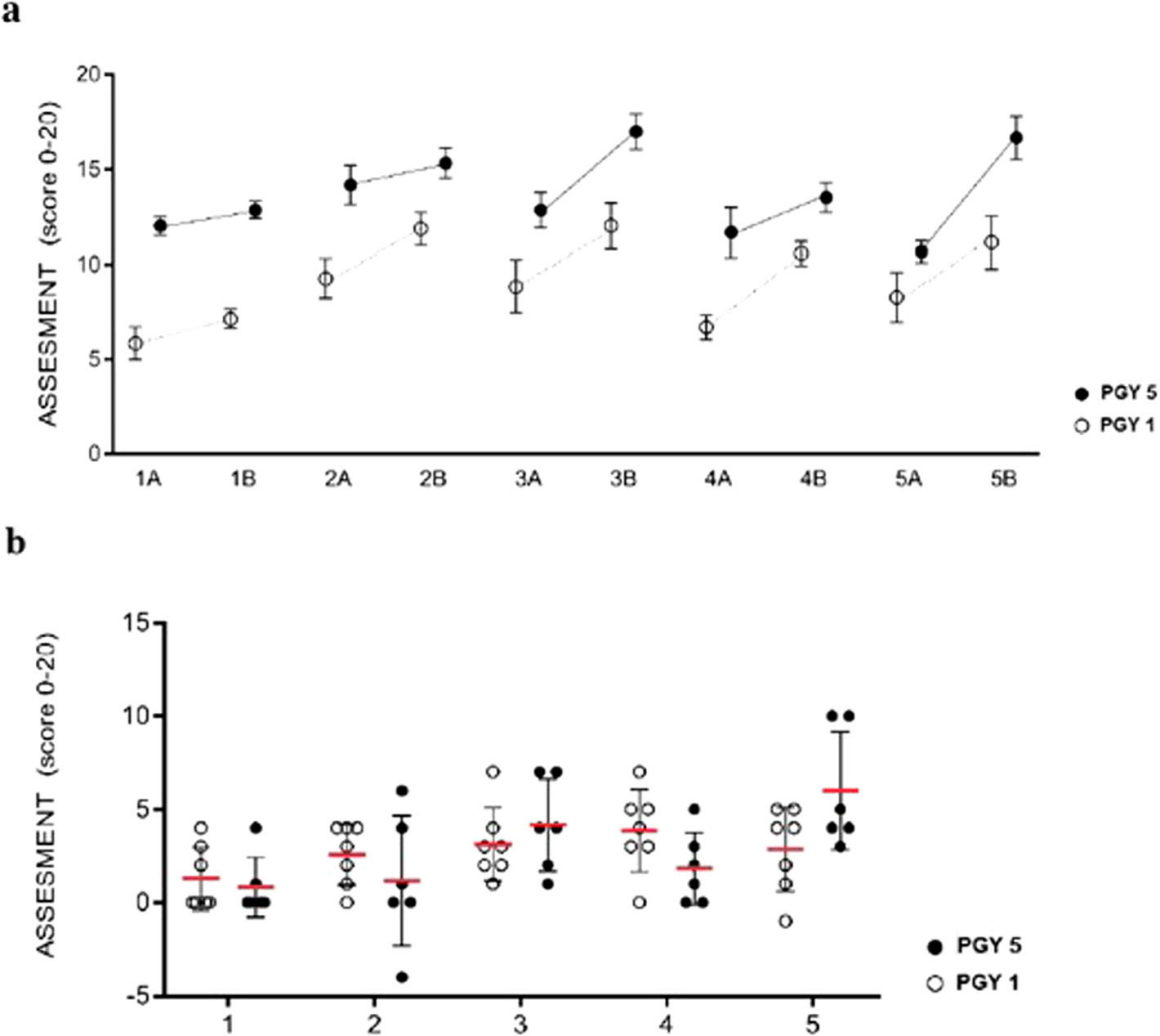
White spots: mean scores before (A) and after (B) simulation training of junior trainees; Black spots: mean scores (A) and after (B) simulation training of senior trainees

The improvement on technical skills is similar in all trainees without statistically significant differences (exercise 1: mean score differences 1.3 vs 0.8; exercise 2: mean score differences 2.6 vs 1.2; exercise 3: mean score differences 3.1 vs 4.1; exercise 4: mean score differences 3.8 vs 1.8; exercise 5: mean score differences 2.8 vs 6) but it is worthy to note that, after the training with simulator, junior residents (PGY 1) present very close scores to what the senior residents do before simulation (PGY 5), especially in exercises 2–4 (Fig. 3).

### Simulation reduces time of execution of the exercises

**Fig. 4:**
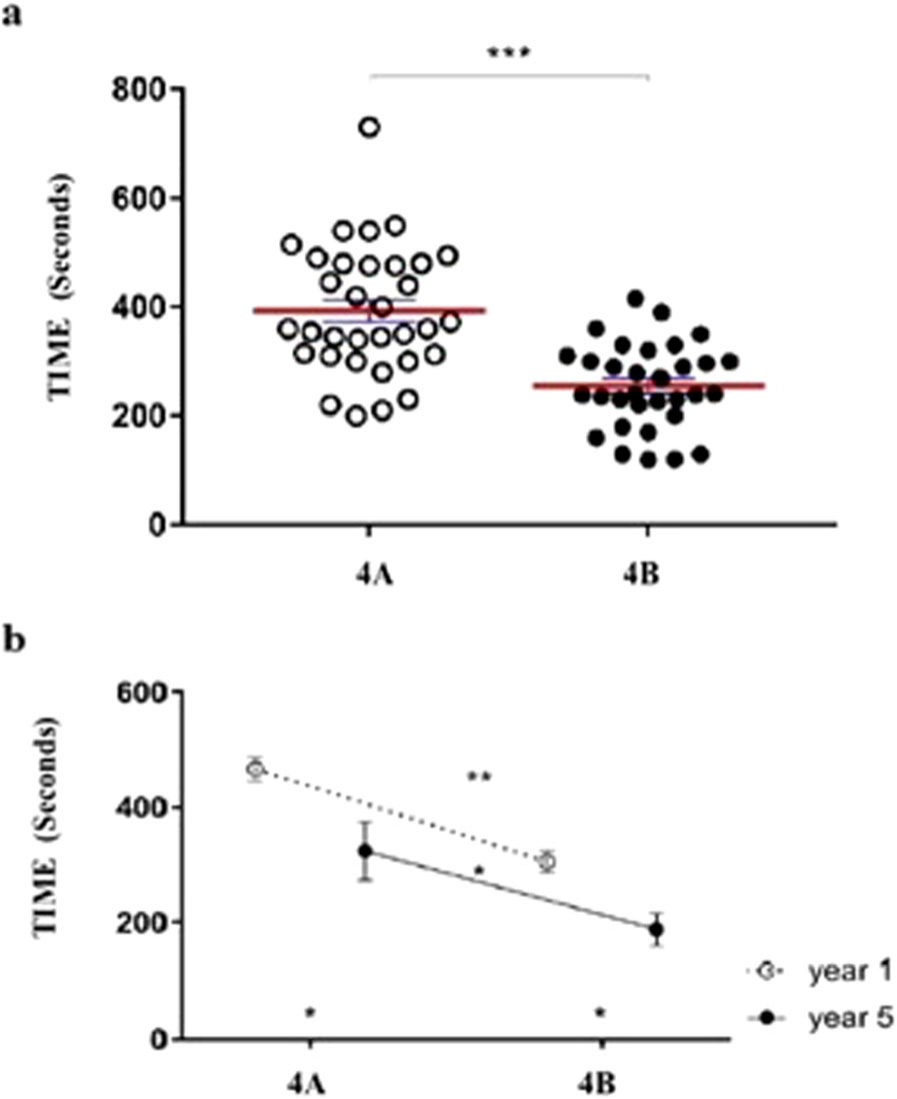
**a**. White spots: time before simulation in exercise 4; Black spots: time after simulation in exercise 4; ***: p value < 0,0001). **b**. White spots: mean times before (A) and after (B) simulation for trainees of the first year; Black spots: mean times before (A) and after (B) simulation for trainees of the last year **: p value 0.0019 *: p value 0.0182)

The time of execution for each task was evaluated exclusively in task number four. For this task, the time was measured for all 33 residents in both the first and second tests. A statistically significant reduction in time in the second test demonstrating how practice affects execution times (Exercise 4a before simulation: mean time 393.5 ± 117.5; Exercise 4a after simulation: mean time 254.8 ± 76.85; *p* < 0.0001) (Fig. 4). It is important to note how the time difference is statistically significant both in the group of residents who are dedicated to surgery and in that who are not dedicated (Mean time in surgery group: 335.4 vs.193.6; p 0.0002; mean time in no surgery group: 436.2 vs. 299.8; *p* < 0.0001). The group with higher experience on surgery performed the entire exercise with shorter times than the no-surgery group, both in the first test and in the second test (Mean time before simulation: 335.4 vs. 436.2; p 0.0029; Mean time after simulation: 193.6 vs. 299.8; p 0.0025). Similar results were also obtained by comparing times of the trainees of first and fifth year, with statistically significant results: in both groups, there was a reduction in the execution time after the simulation (Mean time before simulation: 466 vs. 305.4; p 0.0019; Mean time after simulation: 323.6 vs. 187; p 0.0182), as shown in Fig. 4b.

### Simulation as tool for self-assessment

**Fig. 5:**
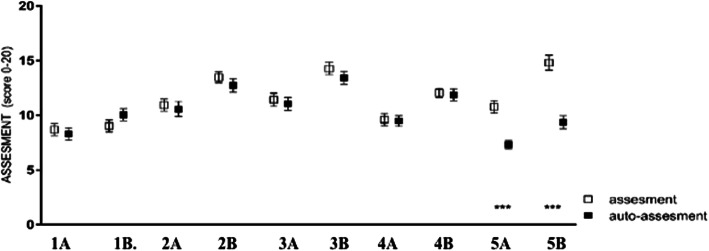
White spots: main scores of assessments; Black spots: main scores of self-assessments; exercise a: before simulation; exercise b: after simulation; ***: p value < 0,0001)

For all the five tasks, both in the first and second tests, assessment and self-assessment were performed. The self-perception of what trainees do is overlapping with the judgment of external observers (Fig. 5). However, this does not occur for the last task, the number five. In this task, the scores of self-assessments are statistically lower than those of evaluation by the external observers. This difference was present before and after simulation training (Exercise 1a: 8.697 vs. 8.303; p 0.9921; Exercise 1b: 9.03 vs. 10.06 p 0.8234; Ex 2a: 10.94 vs. 10.58; p 0.9921; Ex 2b: 13.48 vs. 12.73; p 0.9176; Ex. 3a: 11.42 vs. 11.06; p 0.9921; Ex. 3b: 14.27 vs 13.42; p 0.9050; Ex 4a: 9.606 vs. 9.485; p 0.9921; Ex 4b: 12.06 vs. 11.88, p 0.9921; Ex 5a: 10.79 vs. 7.303; p 0.0001; Ex 5b: 14.82 vs. 9.364; *p* < 0.0001).

### Institutional review board

This study does not require any approval by our IRB according our national regulation about simulation study: all the participants, however, received the informed consent procedure and the participation in the research was completely voluntary. All the results remained confidential and completely anonymity for participants in the manuscript was ensured.

## Discussion

Laparoscopic training is an important aspect of the curriculum for general surgeons, and for gynecologists. This type of procedure requires a set of skills as coordinating instrumentation, cutting, knotting, 2D optics, depth perception which constitute the FSL. These capacities can be reached only with a long and challenging curve of learning in the operating room. And also after these efforts, validated methods for assessing laparoscopic skills remain debatable [12] and there are not approved protocols for the standard use of simulation as a tool for evaluating and improving the surgical skills of the operators [13].

Indeed, some authors have partially questioned the role of simulation as training compared to surgical experience. Comparing the operating times of experienced surgeons with young trainees, despite having similar scores in simulation, the first group presented completely different results in operating room [14].

The aim of this study is not only to provide further data to support the routine use of simulation in the educational field, but, above all, the use of simulation as a useful evaluation tool by an external observer and as a self-assessment test.

In this paper, we demonstrated the role of simulation to evaluate and to improve technical skills in OBGYN trainees. By using OSATS, we evaluated trainees’ training and their improvement, comparing the role of simulation on the learning process of no-expert operators and on meliorating technical skills depending on the prior experience in surgery.

Furthermore, the judgment expressed by external observers corresponds to the trainee’s perceptions. This element is very important to stress. Trainees can self-evaluate their ability to perform FSL and to understand any improvements and this self-assessment corresponds to the judgment of the observer.

Previous researches have demonstrated the usefulness and the validity of OSATS to evaluate residents’ training and their improvement. OSATS have become one of the most used tools for surgical skills assessment, thanks to its construct validity and inter-examiner reliability [11, 15, 16]. Although OSATS were used in laboratory setting, their reliability has been repeatedly demonstrated even in the operating room, making it a very useful instrument to evaluate the operative skills in their entirety [17, 18].

In recent years, however, some authors have shown greater validity and reliability of the global rating scale than the other scores, using it also in a modified form [18]. Also in this study, we used the global rating scale but not in its original form. For each task a specific score has been created, adding criteria like hands synchronization and removing others unnecessary for that exercise.

The use of OSATS to evaluate technical skills in no-general surgery trainees as gynecologists is limited [19]. Even if FSL are all basic technical skills, usually this part of the training is relatively considered. Most of the authors focus their attention on an objective scale for the assessment of specific procedures (hysterectomy, annexectomy, etc) [20].

By using OSATS applied to simulation, we could evaluate different competences of trainees in each task. As expected, young trainees presented lower scores than seniors in statistically significant way. This did not happen for exercise 5, where trainees had to make stitches and knots on a silicon suture-pad. The most reasonable option of this result could be the difficulty of this exercise. In fact, senior trainees still presented better results but not in statistically significant manner.

However, simulation has a pivotal role on ameliorating technical skills. In this study, we demonstrated the use of simulation improves, in a statistically significant way, specific surgical competences as handling instruments, using dominant and no-dominant hands, cutting, making single stitch/knot in a lab setting (Table 1). After simulation, trainees significantly improve their technical skills in all the considered domains. However, it is worthy to note no differences were found in task 1, (introduction of trocars and Verres’s needle). This could be due to different reasons: the system we used for simulation did not perfectly match with reality. In this type of exercise, this lack of likelihood could lead to a difficult evaluation of the correct introduction of trocars and needle. At the same time, for this type of exercise the recognition of the instruments and its handling, outside the operating field, is also intuitive for trainees who have never performed laparoscopy in person. These elements could justify no significant differences before or after simulation.

The benefit of simulation relapsed on junior (first year of residency) and senior (last year) trainees. In each task, competences resulted significantly improved and the powerful of this improvement is particularly important in junior trainees. After simulation, junior trainees presented competences, which are superimposable with those of senior trainees before simulation. This aspect deserves to be highlighted. After a few sessions of simulation, junior trainees reached the same level of basic technical skills on laparoscopic surgery, which senior trainees reached after at the least 2–3 years of work in surgical theater. Also, the execution time improves before and after the simulation training and this difference persists even when comparing junior and senior trainees. This data is in contrast with other papers already published on this issue [21, 14] but it should be emphasized that, in our study, the number of participants is a little bit higher.

Obviously, we must consider this data with extreme caution even because recruited residents were only in Gynecology and Obstetrics. However, this data could support the use of simulation before any training in vivo, on the patient from the beginning of residency.

The development of simulation in Medicine has often been hampered by misperception of reality that would reduce the simulation to a game for adults. In truth, several studies now show how simulation can help not only to acquire technical skills but also to improve the perception of one’s own abilities in that specific task [9, 10, 22]. Several authors even consider mandatory the use of simulation before starting rotation in surgical room [23].

In our study, we have shown that simulation can also be a useful tool for evaluating technical skills of the trainee by an external tutor. This evaluation, moreover, corresponds to the learner’s self-judgment in all the tasks observed except for exercise 5. The discrepancy in this exercise is not causal. This exercise requires high surgical skills and expert operators in clinical practice usually perform it. Evidently, based on this comparison, trainees consider badly their performance even if, from a purely technical point of view, their skills are good at the judgment of their tutors.

This work presents some weaknesses. Evidently, in the learning process, per the Kirkpatrick’s Model, the third level is missing [24]. This aspect is necessary to analyze and evaluate results of training and educational programs.

In fact, this study did not evaluate the differences in the participant’s behavior at work after completing the program.

From an experimental point of view, this study does not have an in vivo “after simulation” evaluation. This limitation of the study depends on several elements, including the decision shared by our Institute’s Ethical Committee, not to test the ability of young trainees in patients for pure research purposes. In our residency program, the approach to the operating room as operators is gradual and takes place in about 5 years. The activity of the first operator in major surgery, where an appropriate laparoscopic skill is required, is reserved for the last 2 years of the program and for those residents who want to specialize themselves in the surgical gynecology.

However, in literature, skills acquired by simulation-based training are transferable to the operative setting [23–25]. The lack of direct access to the operating room by all the specialists limits an adequate training and simulation could help us in this as pointed out by some authors [23–25]. For this reason, we have routinely introduced simulation in our programs.

However, it should be highlighted that the real purpose of the study is to use simulation as a tool for evaluation in safety. In our study, we demonstrated that simulation can be a useful tool to evaluate a trainee by a senior tutor and as self-evaluation.

## Conclusions

Our data support the routine use of simulation in laparoscopy to evaluate and improve surgical skills in trainees. Indeed, trainees could use simulation to self-test their capacity before to practice on patients.

## Data Availability

Yes, data can be requested from the corresponding author.

## Abbreviations

FSL: Fundamental laparoscopic surgery
OSATS: Objective structured assessment of technical skills
PGY: post-graduate training year

## References

[1] Kohn L, Corrigan J, Donaldson M (2000). To Err Is Human: Building a Safer Health System.

[2] Kairys JC, McGuire K, Crawford AG, Yeo CJ (2008). Cumulative operative experience is decreasing during general surgery residency: a worrisome trend for surgical trainees?. J Am Coll Surg.

[3] Agha RA, Fowler AJ (2015). The role and validity of surgical simulation. Int Surg.

[4] Seymour NE, Gallagher AG, Roman SA (2002). Virtual reality training improves operating room performance: results of a randomized, double-blinded study. Ann Surg.

[5] Zendejas B, Brydges R, Hamstra SJ, Cook DA (2013). State of the evidence on simulation-based training for laparoscopic surgery: a systematic review. Ann Surg.

[6] Surgery TABo. ABS to require ACLS, ATLS and FLS for general surgery certification http://www.absurgery.org/default.jsp?news2008.

[7] Hilal Z, Kumpernatz AK, Rezniczek GA, Cetin C, Tempfer-Bentz EK, Tempfer CB (2017). A randomized comparison of video demonstration versus hands-on training of medical students for vacuum delivery using Objective Structured Assessment of Technical Skills (OSATS). Medicine.

[8] Buerkle B, Rueter K, Hefler LA, Tempfer-Bentz EK, Tempfer CB (2013). Objective structured assessment of technical skills (OSATS) evaluation of theoretical versus hands-on training of vaginal breech delivery management: a randomized trial. Eur J Obstet Gynecol Reprod Biol.

[9] Mannella P, Antonelli R, Montt-Guevara M, et al. Simulation of childbirth improves clinical management capacity and self-confidence in medical students. BMJ Simul Technol Enhanced Learn. 2018;4:184–89. 10.1136/bmjstel-2017-000259 PMC8936910 35519004

[10] Mannella P, Palla G, Cuttano A, Boldrini A, Simoncini T (2016). Effect of high-fidelity shoulder dystocia simulation on emergency obstetric skills and crew resource management skills among residents. Int J Gynaecol Obstet.

[11] Martin JA, Regehr G, Reznick R (1997). Objective structured assessment of technical skill (OSATS) for surgical residents. Br J Surg.

[12] Shah J, Darzi A (2001). Surgical skills assessment: an ongoing debate. BJU Int.

[13] Janssens S, Beckmann M, Bonney D (2015). Introducing a laparoscopic simulation training and credentialing program in gynaecology: an observational study. Aust N Z J Obstet Gynaecol.

[14] Crochet P, Agostini A, Knight S, Resseguier N, Berdah S, Aggarwal R (2017). The performance gap for residents in transfer of Intracorporeal suturing skills from box trainer to operating room. J Surg Educ..

[15] van Hove PD, Tuijthof GJ, Verdaasdonk EG, Stassen LP, Dankelman J (2010). Objective assessment of technical surgical skills. Br J Surg.

[16] Goff BA, Lentz GM, Lee D, Fenner D, Morris J, Mandel LS (2001). Development of a bench station objective structured assessment of technical skills. Obstet Gynecol.

[17] Datta V, Bann S, Beard J, Mandalia M, Darzi A (2004). Comparison of bench test evaluations of surgical skill with live operating performance assessments. J Am Coll Surg.

[18] Hopmans CJ, den Hoed PT, van der Laan L (2014). Assessment of surgery residents’ operative skills in the operating theater using a modified objective structured assessment of technical skills (OSATS): a prospective multicenter study. Surgery..

[19] Chang OH, King LP, Modest AM, Hur HC (2016). Developing an objective structured assessment of technical skills for laparoscopic suturing and Intracorporeal knot tying. J Surg Educ.

[20] Knight S, Aggarwal R, Agostini A, Loundou A, Berdah S, Crochet P (2018). Development of an objective assessment tool for total laparoscopic hysterectomy: a Delphi method among experts and evaluation on a virtual reality simulator. PLoS One.

[21] Rao R, Dumon KR, Neylan CJ (2016). Can simulated team tasks be used to improve nontechnical skills in the operating room?. J Surg Educ..

[22] Lathrop A, Winningham B, VandeVusse L (2007). Simulation-based learning for midwives: background and pilot implementation. J Midwifery Womens Health.

[23] Crochet P, Schmitt A, Rambeaud C (2018). Mandatory completion of a box trainer curriculum prior to laparoscopic apprenticeship in the OR for surgical residents: a before and after study. J Gynecol Obstet Hum Reprod.

[24] Donald K. Evaluating Training Programs: The Four Levels: McGraw-hill Education; 1994. Berrett-Koehler Publishers; 1st edition (November 1, 1994).

[25] Sturm LP, Windsor JA, Cosman PH, Cregan P, Hewett PJ, Maddern GJ (2008). A systematic review of skills transfer after surgical simulation training. Ann Surg.

